# Vasogenic cerebral edema associated with the disability in activities of daily living in patients with chronic obstructive pulmonary disease

**DOI:** 10.1002/brb3.1065

**Published:** 2018-07-13

**Authors:** Xiaochuan Wang, Xuqing Huang, Zhongming Gao, Haibo Jiang, Xiaodong Lu

**Affiliations:** ^1^ Department of Neurology The Affiliated Hospital of Hangzhou Normal University Hangzhou China; ^2^ Department of Respiratory Medicine The Affiliated Hospital of Hangzhou Normal University Hangzhou China

**Keywords:** activities of daily living, apparent diffusion coefficient, cerebral edema, COPD, DTI

## Abstract

**Introduction:**

The aim of this study was to explore whether patients with chronic obstructive pulmonary disease (COPD) develop vasogenic cerebral edema, and whether this edema contributes to the COPD‐related disability.

**Methods:**

Eighteen stable patients with COPD and 17 matched healthy volunteers were enrolled. Apparent diffusion coefficient (ADC) values were calculated by voxel‐based analysis using DTI‐Studio software based on diffusion tensor imaging. COPD‐related disability was calculated using activities of daily living (ADL) scale.

**Results:**

In patients with COPD, ADC increased in the white matter fiber tracts including the bilateral anterior cingulum and posterior corpus callosum and in the white matter fibers connecting the bilateral insular cortices, sub‐lobar cortices, and pars triangularis cortices and the left rectus and olfactory gyrus. However, after further controlling for cigarette smoking, the difference in ADC values in the posterior corpus callosum between groups disappeared. Patients with COPD had significantly higher scores in ADL than that in controls. Moreover, ADL scores were positively correlated with the increased regional ADC values.

**Conclusion:**

Vasogenic cerebral edema occurs in patients with COPD. Cigarette smoking may be a risk factor for COPD‐related vasogenic edema. Vasogenic cerebral edema may be related to the COPD‐related ADL impairment.

## 1 INTRODUCTION

The irreversible airflow limitation that is a characteristic of chronic obstructive pulmonary disease (COPD) usually causes arterial oxygen desaturation. Hypoxia can induce the release of vascular endothelial growth factor (Boeck et al., 2015), free radicals (Rossman et al., 2013), and proteolytic enzymes matrix metalloproteinase‐9 (Vlahos, Wark, Anderson, & Bozinovski, 2012). Systemic inflammation is also present in patients with COPD (Furutate et al., 2016). These factors can eventually conspire to increase blood–brain barrier (BBB) permeability (Bailey, Bärtsch, Knauth, & Baumgartner, 2009; Bauer, Burgers, Rabie, & Marti, 2010; Heo, Han, & Lee, 2005; Schoch, Fischer, & Marti, 2002; Stamatovic, Dimitrijevic, Keep, & Andjelkovic, 2006). Nicotine has also been implicated in BBB changes (Paulson et al., 2010). The above data suggest that vasogenic (extracellular) cerebral edema could develop in patients with COPD.

In this study, using voxel‐based analysis (DTI‐Studio software) based on the apparent diffusion coefficient (ADC) map, which was generated from magnetic resonance diffusion tensor images (DTI), we aimed to identify the most relevant DTI biomarker values related to clinical disability in COPD. This method has been used in a previous study that examined patients with schizophrenia, including both first‐episode and chronic patients (Kong et al., 2011). The ADC is sensitive to water diffusion in intracellular compartments and is helpful for distinguishing between types of edema (Schlaug, Siewert, Benfield, Edelman, & Warach, 1997). A decrease in the ADC of the brain suggests a cytotoxic edema, whereas an increase in the ADC of the brain indicates a vasogenic edema (Cernak et al., 2004).

In DTI, ADC and fractional anisotropy (FA) are both used to estimate the tissue integrity. FA, which differs from ADC, measures the overall directionality of water diffusion within cells. A decrease in FA values is associated with cytotoxic brain edema. The ADC and FA show discriminate patterns associated with the development of cerebral edema after hypoxic‐ischemic/reperfusion injury. In hypoxic‐ischemic/reperfusion, it is the ADC but not FA values that correlate with water transport in and out of the cells (Wang, Wang, & Guo, 2012). Compared with changes in FA, quantitative changes in the ADC may be more specific for the prognostication of subtle MRI changes (Mlynash et al., 2010). In addition, technically, ADC values can be directly obtained from ADC maps. Hypoxic brains have been shown to have both cytotoxic edema and vasogenic edema (Bailey et al., 2009; Zhang et al., 2012a), and hypoxia‐related DTI studies usually report both FA and ADC values. However, a previous study only calculated a decrease in FA on DTI maps in patients with COPD (Zhang et al., 2012b). Furthermore, previous studies have reported contradictory results, showing either increased or reduced ADC values in hypoxic brains (Rowland et al., 2017; Rupp et al., 2014).

COPD is associated with a reduction in mobility, inhibiting the performance of the activities necessary for living independently (Katz et al., 2010). Limited mobility is a risk factor for the development of new disabilities in the performance of activities of daily living (ADL; Bernabeu‐Mora et al., 2015). Disability can be identified in each disease stage of COPD (Braido et al., 2011). As the disease worsens, there is a progressive decrease in the ability to perform ADL (Nici et al., 2006). Patients with cerebral edema develop cerebral hypermetabolism (Zetterling et al., 2010), which may increase the energy demand in the brain. Hypermetabolism is linked to disability and cognitive dysfunction (Claassen et al., 2002). Previous studies have demonstrated that white matter (WM) hyperintensity is associated with muscle function (Kilgour, Todd, & Starr, 2014) and that docosahexaenoic acid improves short‐ and long‐term neurological performance as well as reduces cerebral edema (Schober et al., 2016). Therefore, we hypothesized that vasogenic cerebral edema may contribute to the impairment in the performance of ADL in patients with COPD.

## 2 MATERIALS AND METHODS

### 2.1 Subjects

Eighteen patients, who had undergone a period of 30–45 days of in‐hospital rehabilitation following an acute exacerbation of COPD, were enrolled for the present study. Distribution of patients according to Global Initiative for Chronic Obstructive Lung Disease (GOLD) staging category (Pauwels, Buist, Calverley, Jenkins, & Hurd, 2001) is as follows: mild COPD: forced expiratory volume in 1‐second (FEV1)/forced vital capacity (FVC) %<70%, FEV1 > 80% predicted, *n* = 1; moderate COPD: FEV1/FVC<70%, 30%<FEV1 < 80% predicted, *n* = 10; severe COPD: FEV1/FVC<70%, FEV1 < 30% predicted, *n* = 7. At the time of data collection, patients were in clinically stable condition. Seventeen healthy volunteers, with comparable age, gender, and educational background, comprised the control group. Control participants were recruited from the local community. They all had no disability in ADL and no any COPD symptom. All the subjects were free from a known history of cerebrovascular accident, heart failure, neurological disorders, obstructive sleep apnea, coronary artery disease, or diabetes. Patients were provided with therapy including inhalation of ipratropium bromide, bricanyl, ventoline, or budesonide. Demographic and physiological characteristics of the patients and healthy volunteers are listed in Table 1. Procedures were fully explained, and all subjects were provided with a written informed consent before participating in the study. The experimental protocol was approved by the Research Ethics Review Board of Xiamen University.

**Table 1:** Demographic and physiologic characteristics of patients with COPD and healthy volunteers

	COPD patients	Controls	*p* value
Number of subjects (male: female)	18 (14: 4)	17 (13: 4)	–
Age (years)	68.4 ± 7.8	68.1 ± 7.3	0.171
Number of smokers	7	6	–
Number of pack years of smoking	37.9 ± 26.3	29.1 ± 8.0	0.452
Disease duration (years)	7.1 ± 5.2	‐	–
ADL	19.1 ± 4.0	14.9 ± 1.8	<0.001
SaO2 (%)	93.8 ± 4.7	97.2 ± 1.5	0.001
Pulmonary function testing			
VC (% predicted)	63.5 ± 16.8	97.3 ± 14.6	<0.001
FVC (% predicted)	63.6 ± 16.2	100.1 ± 14.9	<0.001
FEV1 (% predicted)	41.8 ± 19.5	98.6 ± 16.3	<0.001

Note. Data are mean ± SD.

ADL: activities of daily living; FEV1: forced expired volume in one‐second; FVC: forced vital capacity; SaO2: arterial oxygen saturation; VC: vital capacity.

### 2.2 Physiological tests

Physiological tests were conducted 1 day before brain images scan. Physiological tests include arterial blood gas analysis and pulmonary function measure. Subjects’ self‐reported ability to perform ADL scale (a modified version of the Lawton and Brody (1969)) was used as ADL score (range 14–56), which includes 14 items assessing current and best prior levels of functioning. Higher score in ADL corresponds to poorer performance and indicates more impaired functions. All data were analyzed using SPSS19.0. Independent t‐test measured between‐group difference. Statistical significance was set at *p *<* *0.05.

### 2.3 Magnetic resonance images (MRI) scan

Images were acquired on a Siemens Trio Tim 3.0T (Erlangen, Germany) at MRI Research Center in Zhongshan Hospital of Xiamen University, China.

A 3D structural MRI was acquired from each subject using a T1‐weighted MPRAGE sequence: TR/TE = 1,900 ms/2.48 ms, FOV = 25 × 25 cm^2^, NEX = 1, matrix = 512 × 256, and slice thickness = 1.0 mm. A DTI pulse sequence with single shot diffusion‐weighted echo planar imaging (TR/TE = 3,600/95 ms, FOV = 24 × 24 cm^2^, NEX = 2, matrix = 128 × 128, and slice thickness = 3 mm) was applied sequentially in 30 noncollinear directions (b‐value = 1,000 s/mm^2^) with one scan without diffusion weighting (b = 0 s/mm^2^). Conventional 2D T1 and T2 images were also acquired for any incidental findings.

### 2.4 ADC analysis

DCM2NII was used to convert diffusion tensor images to the NIFTI format. The images were corrected for distortion induced by head motion and eddy current using FSL (FMRIB Software Library, http://www.fmrib.ox.ac.uk/fsl/). FSL's Brain Extraction Tool was used to remove nonbrain tissue from the images. DTI‐Studio software version 2.4 (http://www.mristudio.org) was then used to calculate parametric maps of ADC mean value and b = 0 images. Each image with no diffusion weighting (b = 0 s/mm^2^) was registered to the individual T1 image and then standardized to MNI152 space using the T2 imaging template supplied with SPM8 to estimate the normalization parameter, which was applied to all the ADC maps, and each voxel was resampled to 2 × 2 × 2 mm. Finally, the normalized ADC maps were smoothed with FWHM of 8 mm.

Two‐sample *t*‐test in SPM8 was used to detect whether each voxel had a higher or lower ADC value in the COPD group compared with the control group. With a liberal cluster threshold of 300 voxels, we took the threshold of *p *<* *0.001 (uncorrected) to define significant group difference, with gender and age as covariates. In addition, with the number of pack years of cigarette smoking as a covariate, we tested the effects of cigarette smoking on ADC values. The pack years of cigarette smoking is a way to measure cigarette smoking intensity. According to Khan et al. (2006), the number of pack years of smoking is calculated as: packs smoked per day×years as a smoker.

### 2.5 Correlation analyses

The ADC value of each subject in different brain regions was extracted using MarsBar toolbox (http://marsbar.sourceforge.net/projects/marsbar), which is implemented in SPM5. Pearson's correlation was used to assess the correlation of regional ADC value and ADL score in patients with COPD with gender, age, FEV1/FVC%, and FEV1% predicted variables. SPSS 16.0 software was used for data analysis. Statistical significance was set at *p *<* *0.05 using Bonferroni's correction for multiple comparisons.

## 3 RESULTS

### 3.1 Physiological findings

Patients with COPD had significantly higher ADL scores and significantly lower arterial oxygen saturation (SaO2), FVC, FEV1, and FEV1/FVC values than that in the controls (Table 1).

### 3.2 ADC values

**Figure 1:**
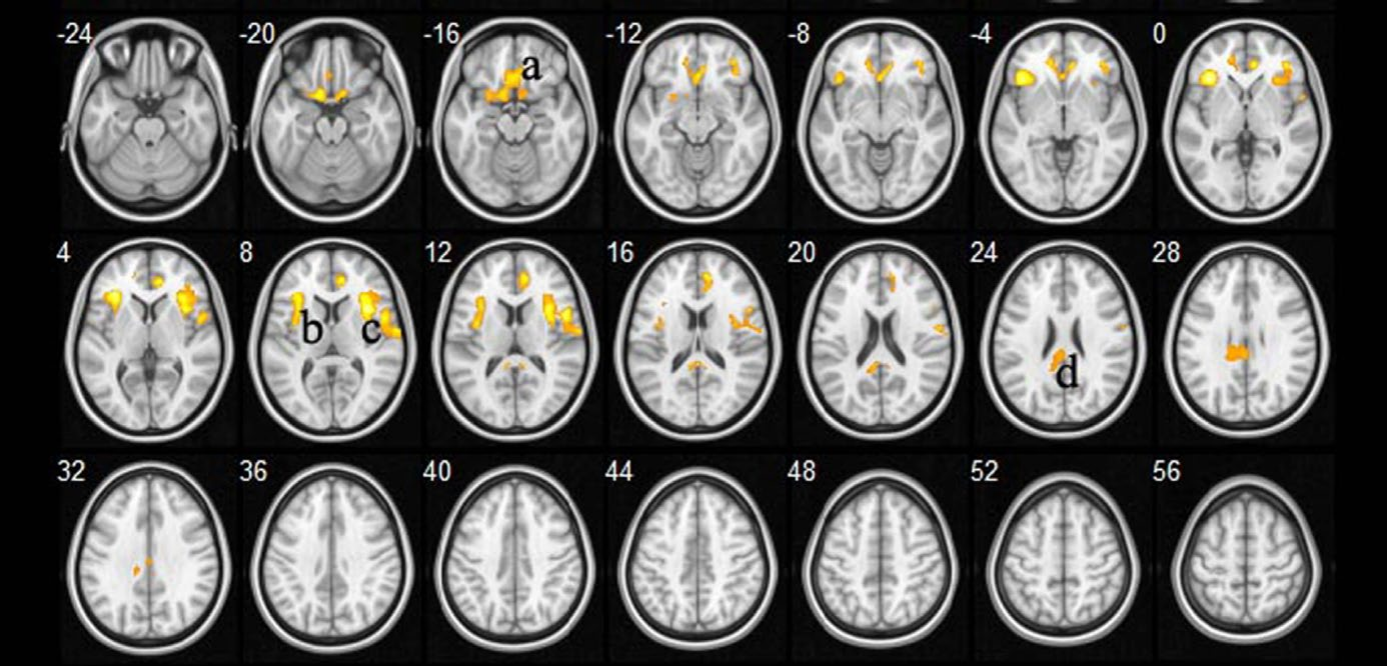
ADC increases in patients with COPD compared with healthy controls, controlling for gender and age (*p *<* *0.001, uncorrected). Statistical map of group comparison of ADC value on a voxel‐wise basis. Detailed information of region a, b, c, and d is shown in Table 2

Whole brain voxel‐wise statistic analysis showed that patients with COPD had significantly increases of ADC in a broad range of brain WM compared with controls, controlling for gender and age. The significantly affected regions included the bilateral anterior cingulum and posterior corpus callosum and the WM fibers connecting the bilateral insular cortices, sublobar cortices, and pars triangularis cortices and the left rectus and olfactory gyrus (Figure 1; Table 2).

**Table 2:** Main regions showing ADC values in patients with COPD compared with healthy controls

Regions		Voxels (mm^3^)	MNI (peak)			ADC(10^−3 ^mm^2^/S)(Mean(SD))		*t* value
			*x*	*y*	*z*	COPD patient	Control	
a	Bilateral anterior cingulum/left rectus/Left olfactory gyrus	1131	12	44	12	1.119 (0.101)	0.947 (0.057)	5.561
b	Right insular cortex/sublobar cortex/pars triangularis	1401	36	14	8	1.081 (0.083)	0.918 (0.049)	7.217
c	Left insular cortex/sublobar cortex/pars triangularis	749	−36	26	0	1.025 (0.058)	0.907 (0.043)	6.913
d	Posterior corpus callosum	380	−6	−42	14	1.484 (0.311)	1.162 (0.172)	4.178

Note. a, b, c, d were indicated in Figure 1.

**Figure 2:**
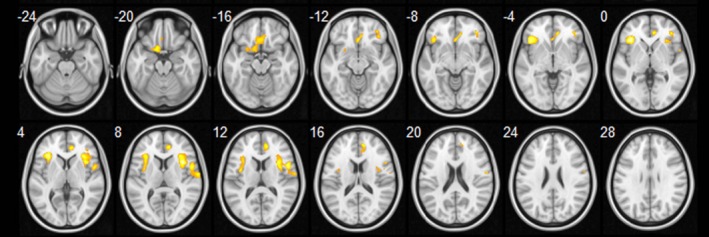
ADC increases in patients with COPD compared with healthy controls, controlling for gender, age, and number of pack years of cigarette smoking (*p *<* *0.001, uncorrected)

However, after further controlling for number of pack years of smoking, the group difference in all regions remained significant except for the posterior corpus callosum (Figure 2).

### 3.3 Correlation of ADL and ADC

**Figure 3:**
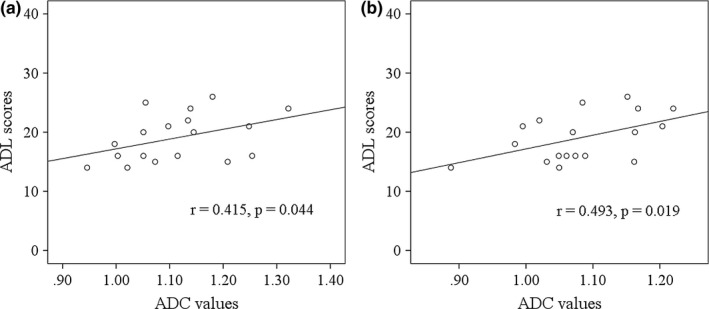
Correlations of ADC values in the brain region (a) and (b) with ADL scores in patients with COPD. Detailed information of region a and b is shown in Figure 1 and Table 2

In patients with COPD, ADC values in the anterior cingulum and WM fibers connecting the right insular cortex, sublobar cortex, and pars triangularis had significant positive correlations with ADL scores (Figure 3).

## 4 DISCUSSION

In the present study, significantly increased ADC values were observed in a number of WM regions in patients with COPD, indicating the occurrence of vasogenic edema. After further controlling for the number of pack years of smoking, the difference in ADC values in the posterior corpus callosum between groups disappeared. Patients with COPD showed poor performance in ADL. Moreover, the regional edema was positively correlated with the performance of ADL.

Consistent with previous findings in patients with COPD, widespread increases in ADC values have also been observed in patients with stroke (Schlaug et al., 1997; Yang & Rosenberg, 2011) and in patients with obstructive sleep apnea syndrome (Emin Akkoyunlu et al., 2013). In a previous study, a decrease in FA values in patients with COPD was observed (Zhang et al., 2012b). Decreased FA values are associated with cytotoxic brain edema. Therefore, both vasogenic and cytotoxic brain edema might develop in hypoxic patients, which are consistent with the findings in populations that are exposed to high‐altitudes (Zhang et al., 2012a).

In a recent study, Cullu et al. (2013) detected higher ADC values in the frontal, temporal, parietal, and occipital deep WM in patients with COPD than in these regions in control participants. There are several differences between our study and the study by Cullu and colleagues, such as (a) the regions of interest (ROI) were selected based on diffusion‐weighted imaging (DWI) in their study, whereas voxel‐based DTI analysis was performed on the whole brain in our study. DTI represents a further development of DWI. In contrast to DWI, DTI measures diffusion ellipsoids using 6 or more gradient directions (Beaulieu & Allen, 1994). Moreover, it is difficult to objectively and reproducibly place ROIs on small or thin tracts on the images of individual subjects, and the ADC value obtained from ROI analysis is largely affected by the location and size of the ROIs used (Giuliani, Calhoun, Pearlson, Francis, & Buchanan, 2005). (b) All the patients in the study by Cullu and colleagues were smokers. Gazdzinski et al. (2005) demonstrated that cigarette smokers exhibit significantly larger temporal and frontal WM volumes than nonsmokers, while Gons et al. (2011) revealed a reduction in the microstructural integrity of the cerebral WM in cigarette smokers. Changes in DTI findings related to smoking behavior have been demonstrated in multiple regions, including the corpus callosum, internal capsule, and prefrontal WM at multiple levels (Hudkins, O'Neill, Tobias, Bartzokis, & London, 2012; Paul et al., 2008; Zhang, Stein, & Hong, 2010). In our study, after controlling for the number of pack years of smoking, there was no observable group difference in the ADC in the posterior corpus callosum. Therefore, we can speculate that the damage in the temporal, parietal, and occipital WM that was revealed by Cullu et al. (2013), and the damage in the WM in areas such as the corpus callosum could be partly caused by nicotine. (c) The examinations of Cullu and colleagues were performed using a 1.5 Tesla MRI scanner. Huisman et al. (2006) studied the “impact” of magnetic field strength on DTI metrics and found that lower ADC values were recorded at higher field strengths, comparing 1.5 with 3.0 Tesla MRI scanners. In summary, the present study gave a more complete and accurate evaluation of microstructural integrity of the cerebral WM in COPD with and without cigarette smoking.

Vasogenic edema is the result of water movement from the vasculature to the brain parenchyma (Donkin & Vink, 2010). Vascular endothelial growth factor (Boeck et al., 2015), free radicals (Rossman et al., 2013), matrix metalloproteinase‐9 (Vlahos et al., 2012), and systemic inflammation (Furutate et al., 2016) have been found to play a role in the modulation of BBB permeability after hypoxic stress in COPD. The vascular endothelial growth factor‐mediated increase in BBB permeability involves nitric oxide‐ and cGMP‐dependent pathways (Schoch et al., 2002). Free radicals can contribute to BBB disruption directly and can also trigger molecular pathways related to the dysfunction of ion transporters in the cell membrane and those related to increased vascular permeability (Heo et al., 2005). Matrix metalloproteinase‐9 mediates vascular leakage in the brain by rearranging tight junction (Bauer et al., 2010). Proinflammatory mediators, such as oxidative mediators, adhesion molecules, cytokines, and chemokines, act directly on brain endothelial cells causing the loosening of the junction complexes between endothelial cells (Stamatovic et al., 2006).

Functional imaging studies have identified the brain regions that are relevant for action sequencing, conceptual and contextual knowledge, and spatiotemporal organization of movements of ADL. The main cortical activation regions for processing conceptual and spatial information for ADL include the ventral frontal cortices and anterior cingulate cortex (Stamatovic, Brandi, Goldenberg, Hughes, & Hermsdörfer, 2014). Several studies have also demonstrated that the ventromedial prefrontal cortex, caudolateral orbitofrontal cortex, insular cortex, and corpus callosum are involved in muscle function (Kilgour et al., 2014), which is critical for ADL. In addition, focal frontal lesions or pathological changes impair ADL (Godbout, Grenier, Braun, & Gagnon, 2005; Marshall, Fairbanks, Tekin, Vinters, & Cummings, 2006). Our present study revealed edema of the WM in the fiber tracts mentioned above or in the fibers connecting these cortices. Moreover, the increase in the ADC values in the WM of these regions was positively correlated with the ADL performance. Therefore, edema of the frontal fibers may contribute to ADL impairment.

There are two limitations in our study. The first limitation is the small number of cases studied. The second limitation is the small number of smokers included in both the COPD and control groups. Therefore, in voxel‐based statistics, with the number of pack years of smoking as a control factor, these smokers might not reflect the populations of patients with COPD, given the small numbers of subjects in our study. Future studies should include a larger sample of patients with COPD with and without cigarette smoking histories to enable a direct comparison between smoking and nonsmoking patients.

## 5 CONCLUSIONS

The increased regional ADC values in the WM in patients with COPD are suggestive of vasogenic cerebral edema. Cigarette smoking may be a risk factor to COPD‐related vasogenic edema. Edema‐induced deficits in spatiotemporal organization and muscle function may contribute to the impairment of ADL in patients with COPD. Our findings support the hypothesis that cerebral edema is involved in the morbidity and mortality associated with hypoxic disease (Donkin & Vink, 2010). ADC values may be a relevant prognostic biomarker for morbidity and mortality. Clinical treatment of brain edema should be considered in patients with COPD. Vasogenic edema may occur after a hypoxic insult in combination with the release of inflammatory mediators. Future studies should clarify the mechanisms underlying vasogenic edema.
